# Assessing Liver and Iron Markers in Steady State Pediatric SCD Patients to Ascertain the Hepatic Consequences of Hemotransfusion: A Case‐Control Study in Ghana

**DOI:** 10.1002/hsr2.72090

**Published:** 2026-03-13

**Authors:** John Agyemang Sah, Stephen Twumasi, Allwell Adofo Ayirebi, Benedict Sackey, Enoch Odame Anto, Richard Vikpebah Duneeh, Ebenezer Senu, Veronica Barnor, David Boakye, Daniel Nii Martey Antonio, Wina Ivy Ofori Boadu

**Affiliations:** ^1^ Department of Medical Diagnostics, Faculty of Allied Health Sciences, College of Health Sciences Kwame Nkrumah University of Science and Technology Kumasi Ghana; ^2^ Kuntanase Government Hospital Bosomtwe Ghana; ^3^ Department of Medical Laboratory Technology, Faculty of Allied Health Sciences Garden City University Kenyase Kumasi Ghana; ^4^ Department of Medical Laboratory Technology, Faculty of Allied Health Sciences Kumasi Technical University Kumasi Ghana; ^5^ Legacy Hospital Kumasi Ghana; ^6^ Medicare College of Applied Sciences Kumasi Ghana; ^7^ School of Medical and Health Sciences Edith Cowan University Perth Australia; ^8^ Centre for Precision Health, ECU Strategic Research Centre Edith Cowan University Perth Australia; ^9^ Department of Medical Laboratory Sciences University of Health and Allied Sciences Ho Ghana; ^10^ Department of Molecular Medicine, School of Medicine and Dentistry Kwame Nkrumah University of Science and Technology Kumasi Ghana; ^11^ Asokwa Children's Hospital Kumasi Ghana

**Keywords:** iron markers, iron stores, liver enzymes, liver function test, sickle cell disease, transfusion

## Abstract

**Background and Aims:**

Between 200,000 and 300,000 children with sickle cell disease (SCD) are born in Africa every year, with 75%–80% of these children living in sub‐Saharan Africa. In newborns with SCD, significant iron accumulations may develop because of their increased risk of requiring multiple blood transfusions. This study aimed to assess the hepatic effects of hemotransfusion in pediatric SCD patients in steady state by measuring liver and iron markers.

**Methods:**

This case‐control research enrolled 120 children with SCD and 60 children without SCD from the Asokwa Children's Hospital Sickle Cell Clinic and Child Welfare Clinic. Participants’ sociodemographic information, history of blood transfusion, and sickle cell genotype were thoroughly documented using a structured questionnaire and patient case records. Venous blood was drawn from each participant for laboratory analysis. Statistical significance was considered at *p* < 0.05.

**Result:**

Serum iron was lowest in HbAA (14.60 [6.20–34.30] µmol/L) and highest in HbSS (24.45 [7.80–51.40] µmol/L; *p* < 0.001). Significant elevated total iron binding capacity, ALT, and GGT levels were observed in children with SCD than in children without SCD. Children with “SS” genotype recorded the highest transfusion history and had significantly elevated levels of serum iron and transferrin saturation than those with genotype “SC” (*p* < 0.05). There were significant correlations between iron markers and ALT (*p* < 0.05). In a linear regression prediction model, an increase in the number of frequency of hemotransfusion among children with SCD resulted in 2.6 µmol/L increase in serum iron levels (*β* = 2.6, *p* < 0.05), 40 ng/mL increase in ferritin levels (*β* = 40, *p* < 0.05), and 8% increase in transferrin saturation (*β* = 8, *p* < 0.05) among children with SCD.

**Conclusion:**

Elevated iron stores and liver enzymes are associated with SCD in children, especially those with a history of transfusion, who should be routinely monitored for elevated iron stores and liver enzymes for early interventions and management.

## 1 Introduction

An inherited abnormality in the structure of hemoglobin causes sickle cell disease (SCD). The beta‐globin chain has valine replacing glutamine at Position 6 in the aberrant hemoglobin [1]. At low oxygen tension, the hemoglobin variant produced polymerizes, resulting in red blood cell sickle malformation and enhanced mechanical fragility, limited life, and chronic hemolytic anemia [1]. Homozygous inheritance of HBS (HbSS) is the most prevalent and severe type of SCD [2]. Coinheritance of HbS with additional mutations of the hemoglobin beta gene, most notably a second structural hemoglobin variant, hemoglobin C (HBC), and β‐thalassemia, are other types of SCD. The worldwide occurrence of SCD is projected to be approximately 300,000–400,000 children annually, with sub‐Saharan Africa bearing the largest proportion. SCD complications account for the mortality of a high number of children under the age of 5, teenagers, and pregnant women. Africa has over 60% of the global carriers of sickle genes [3]. Children with SCD may have persistently low hemoglobin concentrations, normal mean cell volume (MCV), and high mean white cell counts (WBCs), whereas those with sickle cell trait show hematological characteristics similar to those of healthy people [4].

According to Amanor et al., about 17% of steady‐state SCD children had elevated iron stores in a cross‐sectional study conducted at the Komfo Anokye Teaching Hospital, Kumasi, Ghana [5]. This shows that the severity of increased iron storage in children with SCD in the Ashanti Region, Ghana, is particularly substantial, with 2% of newborn babies in Ghana having SCD [6]. It is therefore important to monitor the iron stores of children with SCD and its effect on their organs, such as the heart, the liver, the pancreas, the thymus, and the spleen [5].

Children with SCD demonstrate iron deposition in their organs, such as the liver, due to frequent blood transfusions [7]. Despite chelation therapy, liver injury is common among children with chronic transfusion, and so iron overload is a feared complication of multiple transfusions in children with SCD [8]. Increased serum alanine aminotransferase (ALT) and aspartate aminotransferase (AST) levels successfully predict hepatic injury and may even suggest higher grades of hepatic injury [9].

Increased liver iron concentration (LIC) is found to correspond with increased serum ferritin (SF) level (HIC > 7 mg/g usually corresponds with ferritin level >1000 mg/mL) [10]. Due to poor monitoring of the history of blood transfusion of children with SCD in countries like Ghana, iron overload is usually not recognized because the SF level, which should be measured periodically, is not done [11]. Moreover, there is a limited study on iron stores and liver enzymes among children with SCD. The study's objective was therefore to assess the iron stores of children with SCD and its effect on enzymes that are specific to the liver and to provide documented evidence that can inform decisions on the management of SCD.

## 2 Materials and Methods

### 2.1 Study Design, Duration, and Study Setting

This was a prospective case‐control study conducted at the Asokwa Children's Hospital Sickle Cell Clinic and the Child Welfare Clinic of the Asokwa Children's Hospital. Over a period of 6 months, April to September 2023, data were collected for the study. Asokwa Children's Hospital is located in the Kumasi Metropolis, specifically located in the Asokwa Municipality. Asokwa Municipal District constitutes one of the forty‐three districts in Ghana's Ashanti Region. Asokwa is the administrative center of the municipality, which is located in the central section of the Ashanti Region. The municipality has a land area measuring 25.31 km^2^ (9.77 square miles), a population density of 4964 per kilometer square, a total population of 125,642, and an annual population change of −1.0% (2010–2022). The hospital is the biggest children's facility in the municipality, providing an extensive array of services including emergency medical treatment, laboratory testing, and medical imaging. The medical centre is open around‐the‐clock, all year round.

### 2.2 Ethical Approval and Consent to Participate

The Committee on Human Research Publication and Ethics (CHRPE) of Kwame Nkrumah University of Science and Technology gave ethical authorization for the study with reference: CHRPE/AP/525/21. Permission for approval to use the facilities as the study site was obtained from the Asokwa Children's Hospital. Written informed consent and assent were sought from guardians and the children before the study commenced.

### 2.3 Sample Size Calculation

Sample size was calculated using G*Power software, assuming a medium effect size (Cohen's *d* = 0.5) based on prior studies in Ghana (SCD prevalence of 1.9%) [12], yielding a minimum of 100 cases and 50 controls, adjusted for 20% attrition. To enhance the power of statistics from the study, 120 children with SCD (cases) and 60 children without SCD (controls) were involved in the study.

### 2.4 Study Population

This study recruited 120 children with steady state SCD (cases) and 60 without SCD (controls). To compare severity and transfusion needs, cases were stratified into three hemoglobinopathy groups: HbSS (homozygous, most severe), HbSC (compound heterozygous with HbC), and HbSF (sickle hemoglobin with persistent fetal hemoglobin). Additionally, controls were defined as individuals with a confirmed hemoglobin AA (Hb'AA') phenotype with no history of chronic diseases. All participants were less than 13 years old. Steady state (i.e., no crisis/fever in the past 4 weeks) SCD participants and normal individuals were recruited from the sickle cell clinic and welfare clinic of the Asokwa Children Hospital, respectively. Both cases and controls were without a history of inflammatory conditions, including fever and infections (HIV, Hepatitis B&C). Participants with SCD who demonstrated symptoms of an acute illness (fever or need for an urgent care centre referral), a clinically suspected urinary tract infection, severe hematuria, or symptoms suggestive of a sickle cell pain crisis were also excluded.

### 2.5 Sample Collection

Five milliliters (5 mL) of venous blood was drawn from each participant, out of which 2 mL was kept in a K3 EDTA tube and the remaining 3 mL was placed into serum separator tubes (SST). The samples were placed in an ice pack box and transported to the Central Laboratory of Kwame Nkrumah University of Science and Technology. The samples in the K3 EDTA tubes were used for the estimation of full blood count using a hematology analyzer (XN 500‐Sysmex Corporation, Kobe, Japan), HIV, Hepatitis B and C testing. Following centrifugation of the sample in the SST, serum was separated and stored at −80°C until needed. A commercially available ELISA kit (Melson Shanghai Chemical Ltd, China) was used for the SF, serum iron, total iron‐binding capacity (TIBC), and transferrin saturation. Reagents were used in accordance with the manufacturer's instructions to measure samples from the controls and the participants using the solid phase ELISA technique. ALT, AST, and gamma‐glutamyltransferase (GGT) were analyzed using a fully automated chemistry analyzer.

### 2.6 Data Collection

Socio‐demographic data such as age, sex, and place of residence of study participants were recorded using well‐structured questionnaires and patients' case records. Furthermore, relevant clinical information, such as history of blood transfusion, the hemoglobin genotypes, medication history, other medical conditions, and history of sickle cell crises, was obtained from participants' medical records available on the healthcare database that is the health administration management system used by the Asokwa Children's Hospital.

### 2.7 Laboratory Analysis

The participants’ hemoglobin variants were confirmed via cellulose acetate electrophoresis at pH 8.9, followed by high‐performance liquid chromatography (Bio‐Rad variant II dual program hemoglobin testing), and results were reported as HbSS, HbSC, and HbAA [13]. A fully automated 6‐part hematology analyzer (Sysmex XN 550, Sysmex Corporation, Kobe, Japan) was used for the estimation of hematological parameters (FBC). A commercially available ELISA kit (Melson Shanghai Chemical Ltd, China) was used for SF, serum iron, and total iron binding capacity measurement. The reagent was used in accordance with the manufacturer's instructions to measure samples from the controls and the participants using the solid phase ELISA technique. Elevated iron stores were defined as SF >300 ng/mL according to the literature. The Mindray BS‐240 fully automated chemistry analyzer and an assay kit (Shenzhen Mindray Bio‐Medical Electronics Co., China) were used to estimate the plasma concentration and level of AST, ALT, and GGT in serum samples. Elevated liver enzymes were defined as > 41, > 37, and > 30 U/L for ALT, AST, and GGT levels, respectively.

### 2.8 Statistical Analysis

All collected data were entered, cleaned, and coded in Microsoft Excel 2019. Statistical analyses were done using the Statistical Package for Social Sciences Version 26.0 (Chicago, IL, USA) and the R language for statistical computing [14]. Frequency and percentages were used to represent categorical variables. The distribution of continuous variables was determined by the Shapiro–Wilk test for normality, and nonparametric continuous variables were represented by median and inter‐quartile range. The difference between cases and controls for categorical variables was determined by the Chi‐square test and the nonparametric continuous variables were determined by Mann–Whitney *U*‐test. Moreover, the differences across study groups were determined by the Kruskal–Wallis test and post hoc analyses by the Bonferroni pairwise comparison test. The impacts of research variables were determined using correlation and a linear regression model. A *p*‐value of < 0.05 and 95% confidence interval were deemed statistically significant.

## 3 Results

### 3.1 Sociodemographic and Clinical Characteristics

One hundred and eighty subjects were recruited for the study. Of the 120 cases, most were 6–8 years (32.5%), 3–5 years (30.8%), and 0–2 years (19.2%), whilst most of the controls were 0–2 years (45.0%), 3–5 years, and 6–8 years (15.0%). A significant difference in age group distribution was observed among cases and controls (*p* = 0.0010). Moreover, almost half of SCD patients had history of blood transfusion (49.2%), with majority being hemotransfused two to three times (61.0%), and four to five times (27.1%) within the last 7–12 months (39.0%), more than 12 months (32.2%), and some within 0–6 months (28.8%) compared to non‐sickle cell patients (*p* < 0.0001). All sickle cell patients were on hydroxyurea (100.0%) and also took an iron supplement (ferrous sulfate; 98.3%) compared to non‐sickle cell patients (*p* < 0.0001; Table 1).

**Table 1:** Sociodemographic features of the participants.

Variable	Controls (*n* = 60)	Cases (*n* = 120)	*p*‐value
Age group (years)			**0.0010** a
0–2	27 (45.0)	23 (19.2)	
3–5	18 (30.0)	37 (30.8)	
6–8	9 (15.0)	39 (32.5)	
9–12	6 (10.0)	21 (17.5)	
Sex			0.9160^a^
Male	29 (48.3)	59 (49.2)	
Female	31 (51.7)	61 (50.8)	
History of blood transfusion			**< 0.0001** b
No	60 (100.0)	61 (50.8)	
Yes	0 (0.0)	59 (49.2)	
Number of times of transfusion			n/c
2–3	0 (0.0)	36 (61.0)	
4–5	0 (0.0)	16 (27.1)	
6–8	0 (0.0)	7 (11.9)	
Last transfusion duration (months)			n/c
0–6	0 (0.0)	17 (28.8)	
7–12	0 (0.0)	23 (39.0)	
≥ 13	0 (0.0)	19 (32.2)	
Medication			**n/c**
None	60 (100.0)	0 (0.0)	
Hydroxyurea	0 (0.0)	120 (100.0)	
Iron supplement intake			**< 0.0001** b
No	60 (100.0)	2 (1.7)	
Yes	0 (0.0)	118 (98.3)	
Iron chelating therapy			
No	60 (100)	120 (100)	n/c
Yes	0 (0.0)	0 (0.0)	

*Note:* Data presented as frequencies and percentages. Bold values are statistically significant at *p* value.

^a^ *p*‐values computed by the Chi‐square test.

^b^ *p*‐values computed by Fisher's exact test; n/c, not computed due to constant; bolded *p*‐values mean statistically significant.

### 3.2 Hemtological Profile of the Study Participants

Sickle cell participants had significant lower levels of red blood cells (2.80 × 10^6^), hemoglobin levels (9.20 g/dL), and hematocrit (24.10%) compared to apparently healthy controls (4.17 × 10^6^, 10.30 g/dL, and 29.80%, respectively; *p* < 0.0001). However, sickle cell participants had significantly higher levels of MCV (87.50 µm^3^), MCH (32.65 pg), MCHC (36.40 g/dL), and PLT (270.50 10^3^/µL) compared to those of apparently healthy controls (72.80 µm^3^, 27.70 pg, 34.35 g/dL, and 234.00 10^3^/µL, respectively; *p* < 0.05). Similarly, sickle cell participants had significantly higher levels of RDW‐SD (53.05 µm^3^), RDW‐SD (17.20%), MPV (9.40 µm^3^), and PCT (0.26%) than compared to apparently healthy controls (36.90 µm^3^, 14.15 µm^3^, 9.00%, and 0.22, respectively; *p* < 0.05). Also, lymphocyte count (4.50 10^3^/µL), the counts of eosinophil (0.16 10^3^/µL), and basophil (0.05 10^3^/µL) were significantly higher among sickle cell participants than those of apparently healthy controls (3.36, 0.11, and 0.01 10^3^/µL; *p* < 0.01; Table 2).

**Table 2:** Hemtological characteristics of study participants.

Variable	Controls (*n* = 60)	Cases (*n* = 120)	*p*‐value
WBC (10^3^/µL)	8.55 (6.79–13.95)	9.29 (6.91–12.23)	0.8140
RBC (10^6^/µL)	4.17 (3.62–4.52)	2.80 (2.43–3.39)	**< 0.0001**
HGB (g/dL)	10.30 (9.65–11.08)	9.20 (8.33–10.08)	**< 0.0001**
HCT (%)	29.80 (27.00–32.08)	24.10 (22.25–26.90)	**< 0.0001**
MCV (µm^3^)	72.80 (66.63–78.40)	87.50 (76.15–94.35)	**< 0.0001**
MCH (pg)	27.70 (22.93–27.00)	32.65 (27.88–35.88)	**< 0.0001**
MCHC (g/dL)	34.35 (33.15–35.30)	36.40 (35.33–39.58)	**< 0.0001**
PLT (10^3^/µL)	234.00 (194.25–287.75)	270.50 (201.25–364.75)	**0.0200**
RDW‐SD (µm^3^)	36.90 (35.45–42.13)	53.05 (47.25–61.23)	**< 0.0001**
RDW‐CV (%)	14.15 (12.93–16.10)	17.20 (15.43–19.30)	**< 0.0001**
PDW (µm^3^)	9.55 (8.20–10.60)	9.30 (8.60–10.10)	0.5320
MPV (µm^3^)	9.00 (8.30–9.85)	9.40 (8.80–9.70)	**0.0410**
P‐LCR (%)	17.20 (13.70–23.63)	18.50 (15.10–22.30)	0.2320
PCT (%)	0.22 (0.19–0.28)	0.26 (0.20–0.35)	**0.0060**
NEUT # (10^3^/µL)	3.78 (1.87–6.72)	3.48 (2.21–4.81)	0.2860
LYMP # (10^3^/µL)	3.36 (2.68–4.58)	4.50 (3.13–5.92)	**0.0010**
MONO # (10^3^/µL)	0.79 (0.56–1.27)	0.73 (0.52–1.12)	0.1740
EO # (10^3^/µL)	0.11 (0.01–0.21)	0.16 (0.08–0.32)	**< 0.0001**
BASO # (10^3^/µL)	0.01 (0.01–0.03)	0.05 (0.02–0.07)	**< 0.0001**
NEUT %	44.80 (28.20–59.00)	39.30 (30.90–45.55)	**0.0240**
LYMP %	42.60 (31.63–57.13)	49.25 (42.80–56.88)	**0.0060**
MONO %	8.75 (6.73–12.15)	7.80 (6.50–9.28)	**0.0280**
EO %	1.40 (0.13–1.90)	1.80 (1.00–3.08)	**< 0.0001**
BASO %	0.20 (0.10–0.30)	0.50 (0.30–0.60)	**< 0.0001**

*Note:* Data presented as median and interquartile ranges (IQR); *p*‐values computed by Mann–Whitney *U*‐test; bolded *p*‐values means statistically significant.

Abbreviations: BAS, basophil; EOS, eosinophil; LYMPH, lymphocyte; MCH, mean cell hemoglobin; MCHC, mean cell hemoglobin concentration; MCV, mean cell volume; MON, monocyte; MPV, mean platelet volume; NEUT, neutrophil; PCT‐Plateletcrit; PDW, platelet distribution width; P‐LCR, platelet large cell ratio; PLT, platelet count; RBC, red blood count; RDW‐CV, red cell distribution width‐ coefficient of variation; RDW‐SD, red cell distribution width–standard deviation; WBC, white cell count.

### 3.3 Hemoglobin Genotypic and Hemotransfusion Distribution Among SCD Participants

**Figure 1:**
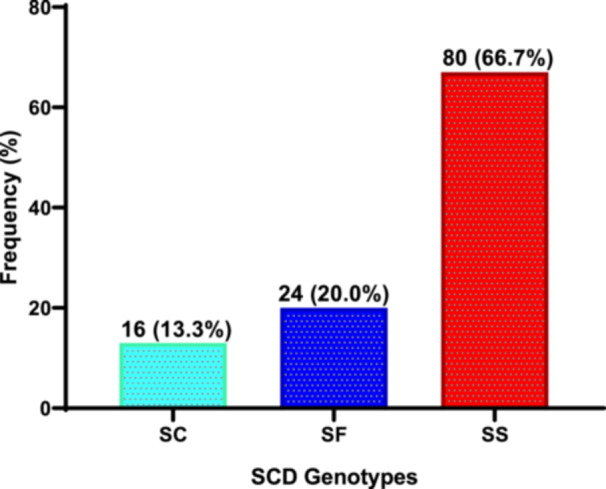
Hemoglobin genotypic distribution among children with SCD. Data presented as frequencies and percentages.

Two‐thirds of sickle cell patients had hemoglobin genotype “SS” (66.7%), whilst 20.0% had “SF” (20.0%) and 13.3% had “SC” (Figure 1). It was observed from the study that out of a total of 59 children with SCD who had history of blood transfusions, 30 (50.8%) of the transfused children with SS genotype had between two and three history of blood transfusions, 11 (18.6%) had between four and five blood transfusions, and six (10.1%) had between six and eight blood transfusions. However, of the transfused children with the SF genotype, five (8.5%) had between two and three history of blood transfusions, three (5.1%) had between four and five blood transfusions, whereas one (1.7%) had between six and eight history of blood transfusions. The children with the SC genotype had the least history of blood transfusions; only one (1.7%) had between two and three blood transfusions, two (3.4%) had between four and five blood transfusions, whereas none of them had between six and eight blood transfusions (Table 3).

**Table 3:** Distribution of blood transfusions among the various genotypes of the children having SCD.

Number of Hemotransfusions	SC (*n* = 3 [5.1%])	SF (*n* = 9 [15.3%])	SS (*n* = 47 [79.6%])
2–3	1 (1.7%)	5 (8.5%)	30 (50.8%)
4–5	2 (3.4%)	3 (5.1%)	11 (18.6%)
6–8	0 (0.0%)	1 (1.7%)	6 (10.2%)

*Note:* Out of the 120 SCD cases, 59 had a history of hemotransfusion.

### 3.4 Correlation Between Iron Markers and Liver Enzyme Levels

Table 4 presents the Spearman's correlation coefficients (*ρ*) assessing the relationship between iron markers and liver enzyme levels (AST, ALT, and GGT). A statistically significant moderate positive correlation was observed between serum iron and ALT (*ρ *= 0.320, *p* < 0.001). Ferritin also showed a weak but statistically significant positive correlation with ALT (*ρ* = 0.159, *p* = 0.034). TIBC was found to have a significant negative correlation with ALT (*ρ* = −0.185, *p* = 0.013), whereas transferrin saturation demonstrated a weak but significant positive correlation with ALT (*ρ* = 0.150, *p* = 0.045). No significant correlations were found between iron markers and AST or GGT, except for serum iron, which showed weak, nonsignificant positive correlations with AST and GGT (*p* > 0.05; Table 4).

**Table 4:** Correlation between Iron markers and liver enzymes.

Iron markers	Liver enzymes	*ρ*	*p*‐value
Serum iron	AST	0.099	0.187
Serum iron	ALT	0.320	**< 0.001**
Serum iron	GGT	0.088	0.237
Ferritin	AST	0.036	0.629
Ferritin	ALT	0.159	**0.034**
Ferritin	GGT	−0.076	0.311
TIBC	AST	−0.020	0.793
TIBC	ALT	−0.185	**0.013**
TIBC	GGT	0.101	0.178
Transferrin saturation	AST	0.082	0.276
Transferrin saturation	ALT	0.150	**0.045**
Transferrin saturation	GGT	0.006	0.936

*Note: ρ*, Spearman's rho correlation coefficient, *p* < 0.05, significant and bolded.

Abbreviations: AST, aspartate transaminase; ALT, alanine transaminase; GGT, gamma‐glutamyl transferase; TIBC, total iron binding capacity.

### 3.5 Comparing Levels of Liver and Iron Markers in Controls (HbAA) and SCD Genotypes (HbSS, HbSC, and HbSF)

A comparison of iron status and liver enzyme parameters across hemoglobin genotypes (HbAA, HbSS, HbSC, and HbSF) was conducted using the Kruskal–Wallis test with Bonferroni‐adjusted post hoc analyses. Serum iron levels showed a statistically significant difference among the hemoglobin groups (*p* < 0.001). Median serum iron was lowest in HbAA (14.60 [6.20–34.30] µmol/L) and highest in HbSS (24.45 [7.80–51.40] µmol/L). Post hoc analysis indicated significant differences between HbAA and each sickle cell group (HbSS, HbSC, and HbSF) and also between HbSS and both HbSC and HbSF. There was no statistically significant difference in SF levels among the groups (*p* = 0.389), although median levels were higher in the HbS genotypes, especially HbSF (279.50 [109.80–506.40] ng/mL). TIBC differed significantly between groups (*p* < 0.001), with lower TIBC in HbAA (85.20 [17.20–158.00] µmol/L) and higher values in HbSS and HbSC. Moreover, transferrin saturation levels were nonsignificantly high in the cases group as compared to the control (*p* = 0.107). Pairwise comparisons revealed significant differences between HbAA and all other groups, as well as between HbSS and both HbSC and HbSF. For liver enzymes, ALT levels were significantly higher in the sickle cell groups compared to HbAA (*p* < 0.001). Median ALT in HbAA was 7.20 (3.70–10.60) U/L versus 13.10–13.80 U/L in the sickle groups. All pairwise comparisons showed statistically significant differences. GGT levels also differed significantly (*p* = 0.034), with elevated medians in HbSS, HbSC, and HbSF relative to HbAA. Significant pairwise differences were noted between HbAA and the sickle cell groups, and between HbSS and HbSC, as well as between HbSC and HbSF. In contrast, AST levels did not differ significantly across hemoglobin genotypes (*p* = 0.262; Table 5).

**Table 5:** Comparing levels of liver and iron markers in controls (HbAA) and SCD genotypes (HbSS, HbSC, and HbSF).

Variable	HbAA	HbSS	HbSC	HbSF	*p*‐values
Serum iron (µmol/L)	14.60 (6.20–34.30)	24.45 (7.80–51.40)	18.35 (8.4–41.3)	21.05 (10.20–51.2)	**< 0.001** ^a^, ^b^, ^c^, ^d^
Ferritin (ng/mL)	208.95 (32.20–397.80)	243.15 (0.00–916.7)	246.05 (71.90–916.70)	279.50 (109.80–506.40)	0.389
TIBC (µmol/L)	85.20 (17.20–158.0)	109.00 (28.80–142.50)	108.75 (28.80–129.50)	94.60 (37.30–138.50)	**< 0.001** ^a^, ^b^, ^c^, ^d^
Transferrin saturation (%)	19.57 (8.49–39.61)	25.07 (8.47–126.04)	17.78 (10.22–31.89)	19.91 (7.94–76.41)	0.107
AST (U/L)	7.05 (2.0–14.0)	7.10 (3.40–17.30)	5.70 (3.50–14.60)	8.60 (3.40–17.30)	0.262
ALT (U/L)	7.20 (3.70–10.60)	13.10 (5.80–29.30)	13.80 (5.80–27.70)	13.65 (5.80–29.30)	**< 0.001** ^a^, ^b^, ^c^, ^d^, ^e^
GGT (U/L)	22.20 (9.50–26.10)	24.55 (6.12–124.60)	24.95 (10.04–121.80)	25.76 (7.30–125.60)	**0.034** ^a^, ^b^, ^c^, ^d^, ^e^

*Note: p*‐values across groups were computed by the Kruskal–Wallis test, and post hoc test was by the Bonferroni pairwise comparison test; *p* < 0.05, significant and bolded.

Abbreviations: ALT, alanine transaminase; AST, aspartate transaminase; GGT, gamma‐glutamyltransferase; TIBC, total iron binding capacity.

^a^ Significant difference between HbAA and HbSS.

^b^ Significant difference between HbAA and HbSC.

^c^ Significant difference between HbSS and HbSC.

^d^ Significant difference between HbSC and HbSF.

^e^ Significant difference between HbAA and HbSF.

### 3.6 Effect of Hemotransfusion Frequency on Iron Stores and Liver Enzymes in SCD Participants

**Figure 2:**
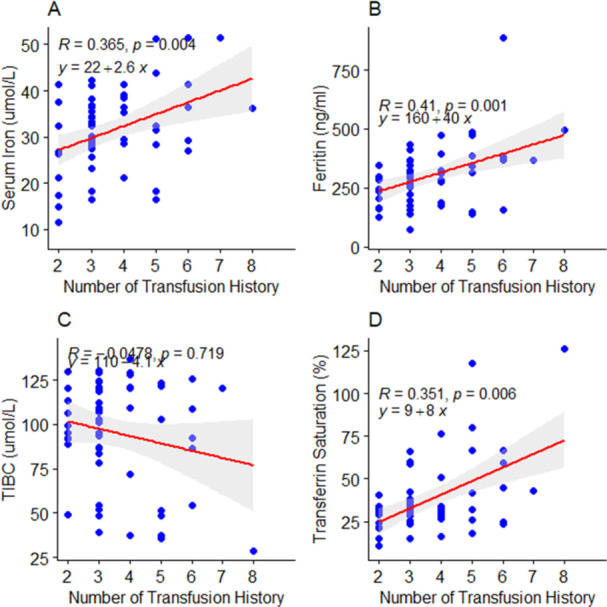
(A–D) Effect of hemotransfusion frequency of children with SCD on iron stores. R, Spearman's correlation value; *y* = *mx* + *c* represents a linear regression equation where variables change respectively. *p* < 0.05: significant.

**Figure 3:**
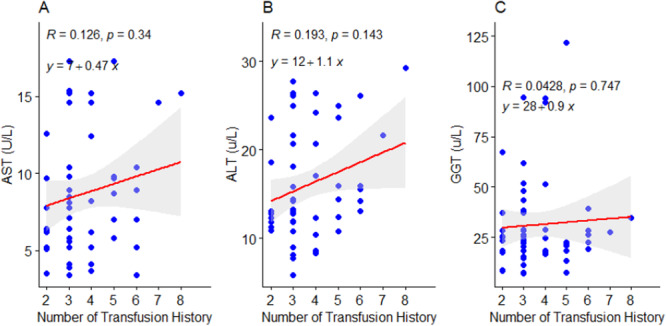
(A–C) Effect of hemotransfusion frequency of children with SCD on liver enzymes. R, Spearman's correlation value; *y* = *mx* + *c* represents a linear regression equation where variables change respectively. *p* < 0.05, significant.

There were significant moderate positive correlations between serum iron (*r* = 0.365, *p* = 0.004), ferritin (*r* = 0.410, *p* = 0.001), transferrin saturation (*r* = 0.351, *p* = 0.006), and hemotransfusion frequency among children with SCD (Figure 2A,B,D). However, an insignificant negative correlation was observed between total iron binding capacity (*r* = −0.0478, *p* = 0.719) and hemotransfusion frequency (Figure 2C). Similarly, there was an insignificant positive correlation between AST (*r* = 0.126, *p* = 0.340), ALT (*r* = 0.193, *p* = 0.143), GGT (*r* = 0.0428, *p* = 0.747), and hemotransfusion frequency among children with SCD (Figure 3A–C). In a linear regression prediction model, an increase in the frequency of hemotransfusion among children with SCD resulted in a 2.6 µmol/L increase in serum iron levels (*β* = 2.6, *p* < 0.05; Figure 2A). Moreover, an increase in the frequency of hemotransfusion resulted in a 40 ng/mL increase in ferritin levels (*β* = 40, *p* < 0.05) among children with SCD (Figure 2B). In addition, an increase in the number of frequencies of hemotransfusion resulted in 8% increase in transferrin saturation (*β* = 8, *p* < 0.05) among children with SCD (Figure 2D).

### 3.7 Comparison of Last Hemotransfusion History, Levels of Iron Stores, and Liver Enzymes

**Figure 4:**
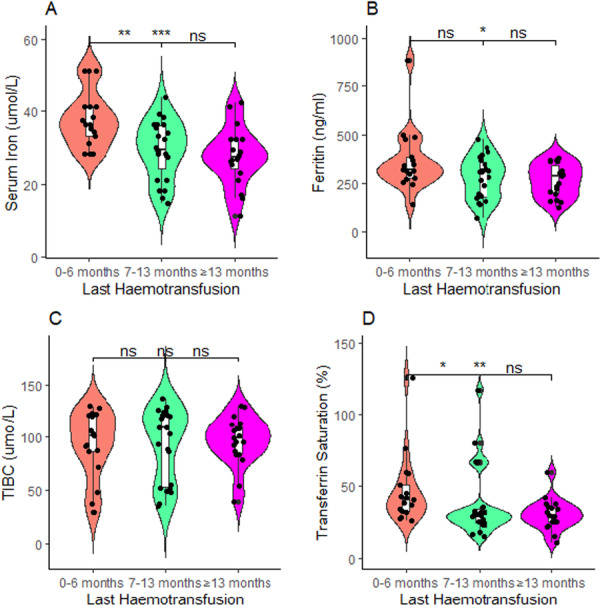
(A–D) Comparison of last hemotransfusion history and levels of iron stores. *p*‐values across groups were computed by the Kruskal–Wallis test, and post hoc test was by the Bonferroni pairwise comparison test; ns, not significant at *p* > 0.05; *p < 0.05; **p < 0.01; ***p < 0.001.

**Figure 5:**
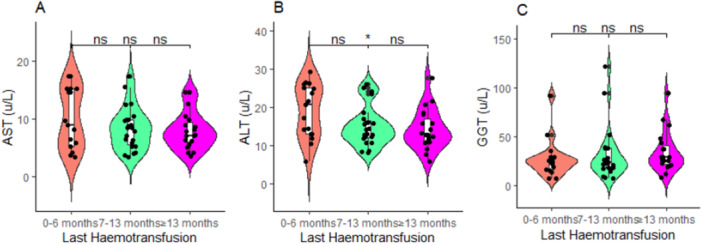
(A–C) Comparison of the last hemotransfusion history and levels of liver enzymes. *p*‐values across groups were computed by the Kruskal–Wallis test, and post hoc test was by the Bonferroni pairwise comparison test; ns, not significant at *p* > 0.05; *p < 0.05.

This study found children with a hemotransfusion history of 0–6 months had significant higher levels of serum iron than children with a hemotransfusion history of 7–13 months (*p* < 0.01), and more than or equal to 13 months (*p* < 0.001; Figure 4A). Also, children with hemotransfusion history of 0–6 months had significant higher levels of ferritin than children with hemotransfusion history of more than or equal to 13 months (*p* < 0.05; Figure 4B). Similarly, children with hemotransfusion history of 0–6 months had significant higher levels of transferrin saturation than compared to children with hemotransfusion history of 7–13 months (*p* < 0.05), and more than or equal to 13 months (*p* < 0.01; Figure 4D). Moreover, children with hemotransfusion history of 0–6 months had significant higher ALT levels than children with hemotransfusion history of more than or equal to 13 months (*p* < 0.05; Figure 5B). Conversely, total iron binding capacity, AST, and GGT were statistically proportional among children with SCD regardless of their hemotransfusion history (Figures 4C and 5A,C).

## 4 Discussion

Over 300,000 newborns are born with SCD each year, with approximately 5% of the world's population being healthy carriers of the SCD gene. Every year, over 200,000–300,000 infants are born in Africa with SCD, with sub‐Saharan Africa containing 75%–80% of these children [15, 16]. Access to iron chelation therapy for the management of transfusion‐related iron overload in Ghana remains limited and challenging, particularly within the context of comprehensive SCD care. While therapies such as deferoxamine, deferiprone, and deferasirox are recognized globally as essential to prevent iron‐related organ damage in chronically transfused patients, these chelators often are not widely accessible or affordable in many parts of sub‐Saharan Africa, including Ghana, due to resource constraints and health system limitations associated with advanced treatment modalities [17]. Children with SCD are more likely to require more blood transfusions, which can lead to elevated iron stores. There is a paucity of data on levels of liver enzymes among children with SCD.

The majority of SCD patients had HBSS, which is consistent with recent studies done by Twumasi et al., which found that almost two‐thirds of steady state SCD patients had HbSS during recruitment in Ghana [13, 18]. Our findings demonstrate significant alterations in iron metabolism and hepatic enzyme markers among individuals with HbSS, HbSC, and HbSF compared to HbAA controls, with the most pronounced changes observed in the HbSS group. This study found that children with SCD had significantly higher serum iron and TIBC levels than apparently healthy controls. This finding is consistent with previous studies indicating that chronic hemolysis, ineffective erythropoiesis, and repeated blood transfusions contribute to iron overload in SCD patients, even in childhood [19, 20]. The significant differences between HbAA and all sickle cell groups reinforce the known impact of abnormal hemoglobin on iron regulation and systemic iron distribution. In akin with this study, Amanor et al. reported that children with SCD had elevated iron stores. However, the levels of ferritin and transferrin saturation were not statistically different between SCD participants and apparently healthy controls. This may be attributed to the wide variability of ferritin levels influenced by factors such as inflammation, liver disease, and transfusion history, all of which are common in this population [19, 21]. Moreover, ferritin is an acute‐phase reactant and may not reliably reflect iron overload in isolation [22]. Similarly, Odunlade et al. reported that SF levels were insignificantly higher in SCD children than those of the controls [23]. In another study, SF correlated with LIC, but was found to be a poor predictor for LIC, leading to an underestimation of LIC by ferritin levels. The SF level of this current study's subjects and that of previous studies are similar, probably due to the fact that the age range of 1–12 years of subjects in the current and previous studies was the same. Akodu et al., in a study of Nigerian children with sickle cell anemia aged 1–5 years, reported a much lower mean SF concentration [24]. Thus, the findings confirm previous suggestions indicating SF levels in infants with SCD vary with age [25]. The age difference between the participants of the various studies is therefore responsible for the disparities. Conversely, TIBC was significantly higher in the HbSS and HbSC groups compared to HbAA, consistent with compensatory upregulation of iron‐binding proteins in the face of elevated iron turnover. Previous studies have shown that TIBC and transferrin saturation vary widely in SCD and may be influenced by nutritional status, erythropoietic demand, and inflammation [26, 27].

Children with SCD had high significant concentration of ALT and GGT compared to children without SCD [28]. In consistent with these study findings, previous study in Nigeria [29] and Ghana [30] also found that serum concentrations of liver enzymes, such as ALT and AST, were significantly higher in people with SCD compared to the controls. The finding of a slight LFT derangement is also consistent with the report by Maher and Mansour [31], which noted that the clinical effects of SCD extend from mild LFT abnormalities in asymptomatic individuals to substantial hepatic abnormal function. Other studies revealed that leukocytosis, fever, and vaso‐occlusive events are all associated with more severe LFT abnormalities [32, 33]. In the current study, SCD patients were all in steady state, hence leukocytosis, fever, and vaso‐occlusive were not explored in participants. AST and ALT play a role in gluconeogenesis by catalyzing the conversion of the amino groups aspartate and alanine and ‐ketoglutaric acid into oxaloacetate and pyruvate, respectively, and glutamate [34]; whilst ALT is mostly located in the liver and kidney, with smaller concentrations also present in the heart and skeletal muscles, AST is present mostly in the heart, liver, skeletal muscle, red blood cells, and kidney [35]. Moreover, high levels of ALT in SCD patients suggest mild hepatocellular injury or hepatic stress, potentially resulting from iron deposition, ischemia‐reperfusion injury, or oxidative damage associated with hemolysis and vaso‐occlusion [36]. Similarly, GGT levels were elevated in sickle genotypes, with statistically significant differences between several groups. Elevated GGT has been linked to cholestasis, hepatic iron overload, and may be an early marker of liver dysfunction in SCD [37, 38]. In contrast, AST levels did not differ significantly across groups. As AST is found in both hepatic and erythrocyte tissues, it may be less specific for liver injury in the context of SCD, where hemolysis contributes substantially to serum AST levels [8, 19, 39, 40]. Overall, these results highlight the complex interplay between hemoglobinopathy, iron metabolism, and liver function. The significant alterations observed in serum iron, TIBC, ALT, and GGT suggest early hepatic involvement in children with SCD, even in the absence of overt clinical symptoms [41]. These findings support the need for routine monitoring of iron parameters and liver function in pediatric SCD patients, especially those undergoing frequent transfusions [19].

The present study examined the correlation between iron markers and liver enzyme levels, with a particular focus on AST, ALT, and GGT. Notably, serum iron, ferritin, TIBC, and transferrin saturation demonstrated significant correlations with ALT, while no significant relationships were observed with AST and GGT. The moderate positive correlation between serum iron and ALT suggests that elevated iron levels may be associated with hepatocellular injury, as ALT is a sensitive marker of liver cell damage. This finding aligns with previous studies indicating that iron overload promotes oxidative stress and lipid peroxidation in hepatocytes, leading to increased ALT levels and liver dysfunction [42, 43]. Similarly, the positive correlation between ferritin and ALT supports the notion that elevated iron storage, particularly in the setting of iron overload, can contribute to liver cell damage [44]. Interestingly, TIBC showed a significant negative correlation with ALT, implying that reduced iron‐binding capacity, often seen in iron overload, may be linked to increased liver enzyme levels. Additionally, transferrin saturation, which reflects the proportion of circulating iron bound to transferrin, was also positively correlated with ALT. These findings are consistent with the literature suggesting that increased transferrin saturation is associated with hepatic iron deposition and elevated liver enzymes [45, 46]. The absence of significant correlations with AST and GGT may be due to their broader tissue distribution and their association with other forms of liver pathology, such as cholestasis or alcohol‐related liver disease (GGT), making ALT a more specific marker for iron‐related hepatocellular injury [47]. Overall, these findings indicate that among the liver enzymes analyzed, ALT, which is liver specific than the other liver enzymes, has the most consistent and statistically significant associations with various iron markers, suggesting a potential link between iron metabolism and hepatocellular integrity [48, 49].

This study observed that SCD children with “SS” genotype recorded the highest transfusion history, and consequently, had higher levels of serum iron and transferrin saturation. This is in line with previous studies depicting predominance of hemotransfusion among children having SCD [23, 50]. Hemotransfusion therapy is currently the standard of care for treating severe conditions like anemia, acute coronary syndrome, and stroke [51]. Iron overload is a dreadful and unavoidable clinical outcome of several transfusions, despite the fact that this treatment significantly lowers the risk of SCD sequelae [11]. The body obtains roughly 200–250 mg of iron with every unit of whole blood transfused [52]. Additionally, persistent hemolysis and increased iron absorption through the digestive tract [53] both result in an excessive release of iron into the body. Similarly, this study found SCD children with a current hemotransfusion history of 0–6 months had significant higher levels of serum iron, transferrin saturation, and ferritin than children with hemotransfusion history of 7–13 months, and more than or equal to 13 months. A study by Amanor et al. also reported that children with SCD who had been given at least three hemotransfusions in the previous 12 months showed higher serum iron and ferritin levels [5]. Approximately 200 mg of iron is delivered into the body after hemotransfusion, with only about 1 mg eliminated daily through the skin and gastrointestinal epithelial layer during hemotransfusion [8]. In hemotransfused SCD patients, iron builds up in the body and also causes an increase in SF levels due to the body's weak physiological system for iron elimination.

Moreover, those with a hemotransfusion history of 0–6 months had significant higher ALT levels than compared to more than or equal to 13 months. According to studies, liver disease is a frequent occurrence in kids with SCD and is a part of the multi‐organ failure that this disorder causes [29]. Due to its complexity, the mechanism of the liver disease in SCD is uncertain [54]. Furthermore, it has been noted that a diseased liver might have a normal size and that liver enlargement does not always indicate disease [55]. As a result, the liver‐related enzyme anomalies discovered in the current investigation could not be attributed to a single component but rather to a number of factors, such as sickle cell occlusion of sinusoids, resulting in hepatic infarction during vaso‐occlusive instances, and red cell sequestration [56, 57].

Patients with SCD are more likely to experience persistent hemolysis, iron recycling, and anemia that necessitates repeated transfusions, leading to elevated iron storage [58, 59]. Consequently, in the current study, there were significant moderate positive correlations between serum iron, ferritin, transferrin saturation, and hemotransfusion frequency among children with SCDs.

Patients who get hemotransfusions of at least 10 units of blood are generally said to be at a high risk of elevated iron storage or iron overload [50]. The results of numerous research nevertheless demonstrate a significant correlation between high iron storage and a sickle cell patient's history of blood transfusion [60, 61, 62]. This agrees with this study's finding that an increase in the frequency of hemotransfusion resulted in a 2.6 µmol/L increase in serum iron levels, 40 ng/mL increase in ferritin levels, and 8% increase in transferrin saturation among children with SCD. Similarly, Amanor et al. also found that a history of chronic hemotransfusion enhanced the chances of having high iron stores and SF concentration [5]. However, Odunlade et al. reported that there was no statistically significant relationship between SF levels and the frequency of blood transfusions [23].

These findings, with 49.2% of cases transfused and *β* = 2.6 µmol/L iron increase per transfusion, underscore the need for national guidelines on iron chelation and routine ferritin/ALT screening in pediatric SCD clinics, potentially reducing hepatic complications in Ghana's ~15,000 annual SCD births.

This study has some limitations. First, dietary iron intake was not assessed, which may have influenced the iron status of participants. Individuals consuming iron‐rich diets may have contributed to the elevated iron levels observed. A noteworthy aspect of this study is that some participants were receiving oral iron supplementation in addition to chronic transfusion therapy. Their inclusion aimed to prevent low hemoglobin levels, iron deficiency or iron deficiency anemia, depleted iron stores, and reduced ferritin concentrations. While this may have influenced iron‐related parameters, it accurately reflects real‐world clinical practice and enhances the external validity and applicability of our findings. A key limitation of this study is that some of the cases and controls were not age‐matched, which may have introduced potential confounding effects and influenced the observed associations. Further research is warranted to investigate the underlying mechanisms and clinical implications of these associations in larger, age‐matched, and more diverse populations. Additionally, we recommend longitudinal studies that incorporate imaging techniques such as magnetic resonance imaging to better assess hepatic iron overload and its potential toxicity in children with SCD.

## 5 Conclusion

Children with SCD exhibit significantly higher levels of serum iron, TIBC, ALT, and GGT compared to children without SCD. Additionally, an increased frequency of hemotransfusion was observed among children with the HbSS genotype. This study further demonstrates that elevated levels of serum iron, ferritin, TIBC, and transferrin saturation are associated with markers of hepatocellular injury, particularly elevated ALT levels. A recent history of hemotransfusion in children with SCD was linked to increased serum iron, ferritin, transferrin saturation, and ALT concentrations. Moreover, a higher number of transfusion episodes was positively associated with increased iron load. These findings underscore the critical need for routine monitoring of iron status and liver enzyme levels in children with SCD, especially those with a history of frequent hemotransfusions. Early identification of iron overload and hepatic dysfunction is essential for timely intervention and effective long‐term management.

## Author Contributions

**Stephen Twumasi:** writing – original draft, writing – review and editing, investigation, conceptualization, methodology. **Enoch Odame Anto:** conceptualization, investigation, writing – original draft, writing – review and editing, software, formal analysis. **Ebenezer Senu:** formal analysis, software. All authors have read and approved the final version of the manuscript.

## Funding

The authors received no specific funding for this work.

## Consent

The authors have nothing to report.

## Conflicts of Interest

The authors declare no conflicts of interest.

## Transparency Statement

The lead author John Agyemang Sah, Stephen Twumasi, and Wina Ivy Ofori Boadu affirms that this manuscript is an honest, accurate, and transparent account of the study being reported; that no important aspects of the study have been omitted; and that any discrepancies from the study as planned (and, if relevant, registered) have been explained.

## Data Availability

The data and materials are available in the corresponding author's institution and will be made available upon formal request. Wina Ivy Ofori Boadu, John Agyeman Sah, and Stephen Twumasi had full access to all of the data in this study and take complete responsibility for the integrity of the data and the accuracy of the data analysis.
